# Exponential *H* ^*∞*^ Synchronization of Chaotic Cryptosystems Using an Improved Genetic Algorithm

**DOI:** 10.1155/2015/640926

**Published:** 2015-07-27

**Authors:** Feng-Hsiag Hsiao

**Affiliations:** Department of Electrical Engineering, National University of Tainan, No. 33, Section 2, Shu Lin Street, Tainan 700, Taiwan

## Abstract

This paper presents a systematic design methodology for neural-network- (NN-) based secure communications in multiple time-delay chaotic (MTDC) systems with optimal *H*_*∞*_ performance and cryptography. On the basis of the Improved Genetic Algorithm (IGA), which is demonstrated to have better performance than that of a traditional GA, a model-based fuzzy controller is then synthesized to stabilize the MTDC systems. A fuzzy controller is synthesized to not only realize the exponential synchronization, but also achieve optimal *H*_*∞*_ performance by minimizing the disturbance attenuation level. Furthermore, the error of the recovered message is stated by using the *n*-shift cipher and key. Finally, a numerical example with simulations is given to demonstrate the effectiveness of our approach.

## 1. Introduction

Stability and stabilization are particularly important factors in time-delay systems, and these factors have, to date, been the focus of many studies. Furthermore, engineering systems [1], such as the structure control of tall buildings, hydraulics, and electronic networks, often involve time delays. Notably, the introduction of a time-delay factor tends to complicate analysis. For this reason, a great deal of research has been focused on developing convenient stability checking methods. The stability criteria of time-delay systems have been traditionally approached from two main directions according to the dependence on the size of the delay. Moreover, since Mackey and Glass [2] first identified chaos phenomena in time-delay systems, time delays have received increasing interest in chaotic systems. Chaotic phenomena have been observed in numerous physical systems and can lead to irregular performance and potentially catastrophic failures [3]. Chaos is a well-known nonlinear phenomenon; it is the seemingly random behavior of a deterministic system characterized by sensitive dependence on initial conditions [4]. Because of these properties, chaos has received a great deal of interest from scientists in various research fields [5, 6]. One particular communication research field, chaotic synchronization, has been extensively investigated.

The chaotic synchronization of identical systems with different initial conditions was first introduced by Pecora and Carroll in 1990 [7]; it aims to lock one chaotic system to another, so that both follow the same path. Based on this concept, various synchronization approaches have been widely developed in the past two decades. Chaotic synchronization can be applied in the vast areas of physics and engineering science and especially in secure communication [8]. The most acceptable synchronization method is the masking method which contains messages in a chaotic system and recovers the original messages from the synchronization [9]. In chaos secure communications, two identical chaotic oscillators called transmitter (master) and receiver (slave) are required. Consequently, chaotic synchronization has become a popular study [10, 11]. However, most synchronization methods are focused on synchronizing two identical chaotic systems with different initial conditions [12]. In fact, experimental and even real systems are often not fully identical; in particular, there are mismatches in the parameters of the systems [12].

In general, there will always be some noise or disturbances that may cause instability. An external disturbance will negatively affect the performance of chaotic systems. Therefore, how to reduce the effect of external disturbances in the synchronization process is an important issue for chaotic systems [13, 14]. The *H*_  _^*∞*^ control has been conferred for synchronization in chaotic systems over the last few years [13–16], and the *H*_  _^*∞*^ synchronization problem for time-delay chaotic systems has been extensively investigated (see, e.g., [14, 17–19]). Accordingly, the purpose of this study is to realize the exponential synchronization of identical multiple time-delay chaotic (MTDC) systems and to simultaneously attenuate the effect of external disturbances on the control performance to a minimum level.

Due to the unique merits in solving complex nonlinear system identification and control problems, neural-network-based modeling has become an active research field in the past few years. Neural networks (NN) consist of simple elements operating in parallel; these elements are inspired by biological nervous systems. As in nature, the connections between elements largely determine the network function. A neural network can be trained to perform a particular function by adjusting the values of the connections (weights) between elements. Therefore, a nonlinear system can be approximated as closely as desired by an NN model via repetitive training (Figure 3). Some examples of successful applications of NN in recent years can be found in [20–25]. For instance, Limanond et al. [20] applied neural networks to optimal etch time control design for a reactive ion etching process. Enns and Si [22] advanced an NN-based approximate dynamic programming control mechanism for helicopter flight control. Despite several promising empirical results and their nonlinear mapping approximation properties, the rigorous closed-loop stability results for systems using NN-based controllers are still difficult to establish. Therefore, an LDI state-space representation was introduced to deal with the stability analysis of NN models [26].

In the past few years, much research effort has been devoted to fuzzy control, which has attracted a great deal of attention from both the academia and industry, and it has been successfully used in wide variety of applications. For example, Wang et al. [27] presented a new measurement system that comprises a model-based fuzzy logic controller, an arterial tonometer, and a micro syringe device for the noninvasive monitoring of the continuous blood pressure wave form in the radial artery. A good tracking performance control scheme, a hybrid fuzzy neural-network control for nonlinear motor-toggle servomechanisms, was provided by Wai [28]; Hwang et al. [29] developed a network-based fuzzy decentralized sliding-mode control for the trajectory tracking of a car-like mobile robot; a hybrid fuzzy-PI speed controller for permanent magnet synchronous motors was proposed by Sant [30]; and Spatti et al. [31] introduced a fuzzy control strategy for voltage regulation in electric power distribution systems: this real-time controller would act on power transformers equipped with underload tap changers.

Despite the successes of fuzzy control, it still has many basic problems that have yet to be solved. Stability analysis and systematic design are certainly among the most important issues for fuzzy control systems. Recently, significant research efforts have been devoted to these issues [32, 33]. All of these studies, however, ignored the modeling errors between nonlinear systems and fuzzy models. In fact, the existence of modeling errors may be a potential source of instability for control designs based on the assumption that the fuzzy model exactly matches the nonlinear plant [34]. In recent years, novel approaches to overcome the influence of modeling errors in the field of model-based fuzzy control for nonlinear systems have been offered by Kiriakidis [34], Chen et al. [35], and Cao and Frank [36].

The genetic algorithm (GA) is a global optimal search tool. By working with a population of solutions, the GA can seek many local minima and thus increase the likelihood of finding the global minimum [37]. The GA employs the Darwinian survival-of-the-fittest theory to yield better characteristics for the individuals in a population and to perform a random information exchange to produce superior individuals [38]. During the evolution, GAs work with a population of individuals called chromosomes represented by binary or real-numbered strings and modify the population through three genetic operations: (1) reproduction, (2) crossover, and (3) mutations. The modified new population is called offspring. The crossover operation is used to combine the information of the selected chromosomes (parents) and generate the offspring. The mutation operation is used to change the offspring genes [39]. Recently, numerous reports on the success of GA applications in control systems have appeared in the literature (see [40–42]). For instance, Navale and Nelson [41] developed an adaptive fuzzy logic controller and made use of GAs to improve the fuzzy rule matrix and fuzzy membership functions for the air handling unit in heating, ventilating, and air conditioning systems. Martínez et al. [40] developed a tracking controller for the dynamic model of a unicycle mobile robot by integrating a kinematic and a torque controller based on type-2 fuzzy logic theory and GAs. Kim and Kang [42] proposed a multiobjective optimal fuzzy control system for the building structure response reduction of a wind-excited tall building by using multiobjective GAs. In this paper, the Improved Genetic Algorithm (IGA) is adopted due to its better performance compared to that of a traditional GA [39, 43].

Cryptography has always been very important in military and business applications for maintaining the secrecy of messages and to prevent information tampering and eavesdropping. This is especially true since the number of transactions being made via the Internet continues to increase at a pace [44]. In this regard, a direct solution to protect messages is to use symmetric encryption. Symmetric encryption uses the same key for both encryption and decryption [45]. There are two basic types of symmetric encryption algorithms, the Data Encryption Standard (DES) and the Advanced Encryption Standard (AES) algorithms [46]. There have been numerous recent reports on the success of symmetric encryption [47–50]. Therefore, the security problem of master-slave systems based on chaotic circuits needs to develop a high secure communication system, which is the other subject of this study.

Almost all existing research on controlling chaos has used fuzzy models to approximate chaotic systems. Although using fuzzy models to approximate the chaotic systems is simpler than using neural networks (NN), the NN models better approach the chaotic systems by iterative training and weight adjustment. That is to say, NN models will have fewer modeling errors which will be much less than fuzzy models. In addition, this study combines the concepts of chaotic synchronization and cryptography to achieve a more secure communication system. First, we use the *n*-shift cipher and key to the original message of transmission for encryption. The encrypted message is reencrypted using chaotic synchronization. Consequently, an effective method is proposed via a neural-network- (NN-) based technique to realize the optimal *H* ^*∞*^ exponential synchronization of multiple time-delay chaotic (MTDC) systems, so that the trajectories of slave systems can approach those of master systems and the effect of external disturbance on control performance is attenuated to a minimum level. The MTDC systems are first approximated by the NN model approach. Next, in terms of Lyapunov's direct method, a delay-dependent criterion is derived to guarantee the exponential stability of the error system between the master and the slave systems. Subsequently, the stability conditions are reformulated into linear matrix inequalities (LMIs). On the basis of the LMIs, a model-based fuzzy controller is then synthesized to stabilize the MTDC systems. Because of the GA capability in random search for global optimization, the lower bound and upper bound of the search space can be set so that the GA will seek better feedback gains of fuzzy controllers in order to speed up the synchronization based on the feedback gains via LMI-based approach. Furthermore, the Improved Genetic Algorithm (IGA) is adopted due to its better performance compared to that of a traditional GA. Based on the IGA, a fuzzy controller is synthesized not only to realize the exponential synchronization but also to achieve optimal *H* ^*∞*^ performance by minimizing the disturbance attenuation level. Finally, the error of the recovered message is stated using the *n*-shift cipher and key.

The remainder of this paper is organized as follows. In Section 2, we establish NN models representing chaotic systems and a model-based fuzzy controller. In Section 3, a robust fuzzy control design is proposed to realize the exponential optimal *H* ^*∞*^ synchronization. The design algorithm is shown in Section 4. In Section 5, the effectiveness of the proposed approach is illustrated by a numerical simulation. Finally, the paper is concluded in Section 6.

## 2. Problem Formulation

Consider two multiple time-delay chaotic (MTDC) systems in master-slave configuration. The dynamics of the master system (*N*_*m*_) and slave system (*N*_*s*_) are described as follows:

(1) Nm: X˙t=fXt+∑k=1gHkXt−τkYt=fXt,

(2) Ns: X^˙t=f^X^t+∑k=1gH^kX^t−τk+BUt+DtY^t=f^X^t,

where *f*(·), $\hat{f}(\cdot )$ , *H*_*k*_(·), and ${\hat{H}}_{k}(\cdot )$ are the nonlinear vector-valued functions, *τ*_*k*_ (*k* = 1,2,…, *g*) are the time delays, *U*(*t*) is the control output, and *D*(*t*) denotes the external disturbance.

In this section, we first use the *n*-shift cipher and key to the original message of transmission for encryption. The encrypted message is reencrypted by using chaotic synchronization. A neural network (NN) model is then established to approximate the MTDC system. The dynamics of the NN model are then converted into a linear differential inclusion (LDI) state-space representation. Finally, based on the LDI state-space representation, a fuzzy controller is synthesized to realize the synchronization of the MTDC systems.

### 2.1. Chaotic Cryptosystem

**Figure 1:**
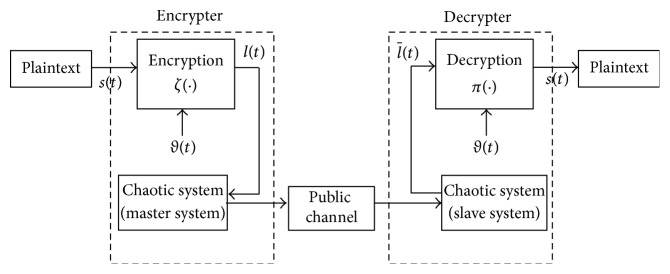
Block diagram of the chaotic synchronization cryptosystem.

A chaotic synchronization cryptosystem is shown in Figure 1. It consists of the encrypter (the master system and an encryption function *ζ*(·)) and decrypter (the slave system and a decryption function *π*(·)). First, the message *s*(*t*) and encryption key *ϑ*(*t*) form an encrypted message *ι*(*t*) via an encryption function. The encrypted message *ι*(*t*) is then combined in the master system. When the chaotic systems are synchronized in the decrypter and encrypter, we can obtain the message $\overset{-}{\iota }(t)$ in the encrypter. Next, the message $\overset{-}{\iota }(t)$ can be decrypted by decryption key *ϑ*(*t*) in the decryption function *π*(·). A decryption function is then used to reveal the message.

We use an *n*-shift cipher for encryption [47]. The *n*-shift cipher is defined by

(3) ιtζst=F⋯FF︸nst,ϑt,ϑt,…,ϑt︸n,

where *h* is chosen such that message *s*(*t*) and encryption key *ϑ*(*t*) lie within (−*h*, *h*). Here, *ι*(*t*) denotes the encrypted signal, and *F*(·) is the following nonlinear function:

(4) Fst,ϑt=st+ϑt+2h,−2h≤st+ϑt≤−hst+ϑt,−h<st+ϑt<hst+ϑt−2h,h≤st+ϑt≤2h.

**Figure 2:**
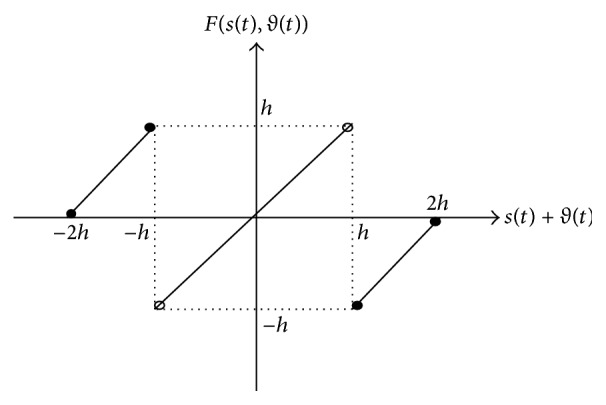
Nonlinear function used in continuous shift cipher.

**Figure 3:**
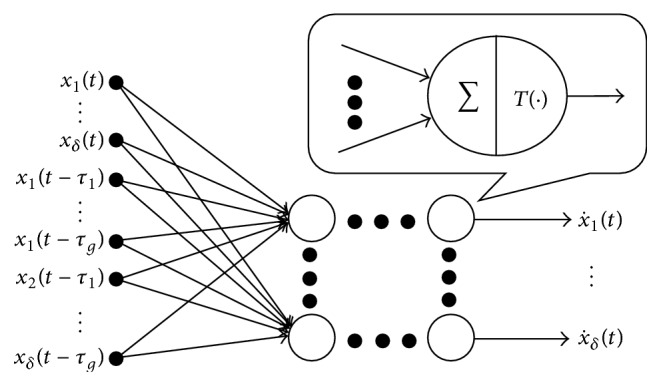
An NN model.

This function is shown in Figure 2.

The corresponding decryption function is the same as the encryption function

(5) st=πι−t=F⋯FF︸nζι−t,−ϑt,−ϑt,…,−ϑt︸n,

where $\overset{-}{\iota }(t)$ is the recovered decryption signal, and *π*(·) is the decryption function. In the *n*-shift cipher, the key signal *ϑ*(*t*) is used *n* times to encrypt the plain signal.

The encrypted message *ι*(*t*) is then combined in the master system. The dynamics of the master system (*N*_*m*_) and slave system (*N*_*s*_) are then described as follows:

(6) Nm: X˙t=fXt+∑k=1gHkXt−τk+BSιtYt=fXt+Sιt,

(7) Ns: X^˙t=f^X^t+∑k=1gH^kX^t−τk+BUt+DtY^t=f^X^t.

### 2.2. Neural-Network (NN) Model

The MTDC system can be approximated by an NN model, as shown in Figure 1, that has *S* layers with *J* ^*σ*^ (*σ* = 1,2,…, *S*) neurons for each layer, in which *x*_1_(*t*) ~ *x*_*δ*_(*t*) are the state variables and *x*_1_(*t* − *τ*_1_) ~ *x*_1_(*t* − *τ*_*g*_), *x*_2_(*t* − *τ*_1_) ~ *x*_*δ*_(*t* − *τ*_*g*_) are the state variables with delays.

Superscripts are used to distinguish the layers and the number of the layers as a superscript to the names for each of these variables. Thus, the weight matrix for the *σ*th layer is written as *W* ^*σ*^. Moreover, it is assumed that *v*_*ς*_^*σ*^(*t*)  (*ς* = 1,2,…, *J* ^*σ*^; *σ* = 1,2,…, *S*) is the net input and *T*(*v*_*ς*_^*σ*^(*t*)) is the transfer function of the neuron. Subsequently, the transfer function vector of the *σ*th layer is defined as

(8) Ψσvςσt≡Tv1σt Tv2σt ⋯ TvJσσtT,σ=1,2,…,S,

where *T*(*v*_*ς*_^*σ*^(*t*))  (*ς* = 1,2,…, *J* ^*σ*^) is the transfer function of the *ς*th neuron. The final output of NN model can then be inferred as follows:

(9) X˙t=ΨSWSΨS−1WS−1ΨS−2⋯Ψ2W2Ψ1W1Λt⋯,

where

(10) $$\begin{array}{c} \Lambda^{T}\left(\;t\;\right)=\left[\begin{array}{cc} X^{T}\left(t\right) & X^{T}\left(t-\tau_{k}\right) \end{array}\right] \end{array}$$

with

(11) Xt=x1tx2t⋯xδtT,Xt−τk=x1t−τ1⋯x1t−τgx2t−τg⋯xδt−τmTfor  k=1,2,…,g.

### 2.3. Linear Differential Inclusion (LDI)

To deal with the synchronization problem of MTDC systems, this study establishes the following LDI state-space representation for the dynamics of the NN model, described as [26, 51]

(12) O˙t=AatOt,Aat=∑i=1ϕhiatA~i,

where *ϕ* is a positive integer, *a*(*t*) is a vector signifying the dependence of *h*_*i*_(·) on its elements, ${\tilde{A}}_{i}\;\;(i=1,2,\ldots ,\phi )$ are constant matrices, and *O*(*t*) = [*o*_1_(*t*) *o*_2_(*t*) ⋯ *o*_*ℵ*_(*t*)]^*T*^. Furthermore, it is assumed that *h*_*i*_(*a*(*t*)) ≥ 0 and ∑_*i*=1_^*ϕ*^ *h*_*i*_(*a*(*t*)) = 1. Based on the properties of LDI, without loss of generality, we can use *h*_*i*_(*t*) instead of *h*_*i*_(*a*(*t*)). The following procedure represents the dynamics of the NN model (4) by LDI state-space representation [26].

First, the output *T*(*v*_*ς*_^*σ*^(*t*)) satisfies

(13) gς0σvςσtTvςσt≤gς1σvςσt,vςσt≥0,gς1σvςσtTvςσt≤gς0σvςσt,vςσt<0,

where *g*_*ς*0_^*σ*^ and *g*_*ς*1_^*σ*^ denote the minimum and the maximum of the derivative of *T*(*v*_*ς*_^*σ*^(*t*)), respectively, and are given in the following:

(14) $$\begin{array}{c} g_{\varsigma \varphi }^{\sigma }=\left\{\begin{array}{cc} \underset{v}{\min}\frac{dT\left(v_{\varsigma }^{\sigma }\left(t\right)\right)}{dv_{\varsigma }^{\sigma }\left(t\right)} & when\;\;\varphi =0\\ \underset{v}{\max}\frac{dT\left(v_{\varsigma }^{\sigma }\left(t\right)\right)}{dv_{\varsigma }^{\sigma }\left(t\right)} & when\;\;\varphi =1. \end{array}\right. \end{array}$$

Then, the min-max matrix *G* ^*σ*^ of the *σ*th layer is defined as follows:

(15) $$\begin{array}{c} G^{\sigma }\equiv \mathrm{diag}\left[\;{g_{\varsigma }^{\sigma }}_{\varphi_{\varsigma }}\;\right]=\left[\begin{array}{ccccc} {g_{1}^{\sigma }}_{\varphi_{1}} & 0 & 0 & \cdots & 0\\ 0 & {g_{2}^{\sigma }}_{\varphi_{2}} & 0 & \ddots & 0\\ 0 & 0 & {g_{3}^{\sigma }}_{\varphi_{3}} & 0 & \vdots \\ \vdots & \ddots & 0 & \ddots & 0\\ 0 & 0 & \cdots & 0 & {g_{J^{\sigma }}^{\sigma }}_{\varphi_{J}} \end{array}\right]. \end{array}$$

Based on the interpolation method, the transfer function *T*(*v*_*ς*_^*σ*^(*t*)) can be represented as follows [26]:

(16) Tvςσthς0σtgς0σ+hς1σtgς1σvςσt=∑φ=01hςφσtgςφσvςσt,

where the interpolation coefficients *h*_*ςφ*_^*σ*^(*t*)∈[0,1] and ∑_*φ*=0_^1^ *h*_*ςφ*_^*σ*^(*t*) = 1. Equations (8) and (16) show that

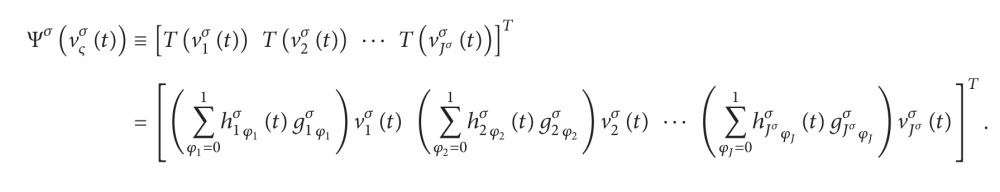

(17)
Therefore, the final output of the NN model (9) can be reformulated as follows:

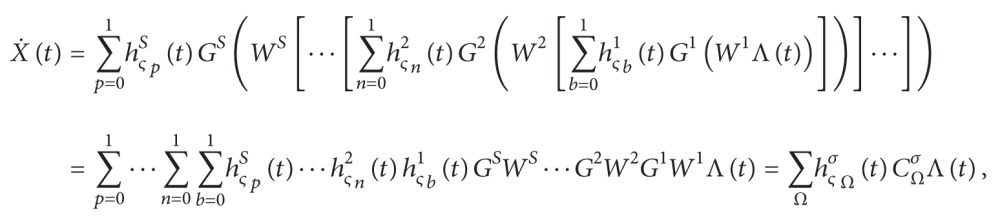

(18)
where

(19) ∑b=01hς1bt≡∑b1=01h11b1t∑b2=01h21b2t⋯∑bJ=01hJ11bJt∑n=01hς2nt≡∑n1=01h12n1t∑n2=01h22n2t⋯∑nJ=01hJ22nJt⋮∑p=01hςSpt≡∑p1=01h1Sp1t∑p2=01h2Sp2t⋯∑pJ=01hJSSpJt∑ΩhςσΩt≡∑p=01⋯∑n=01 ∑b=01hςSpt⋯hς2nthς1bt,ς=1,2,…,Jσ;CΩσ≡GSWS⋯G2W2G1W1

and *b*_*ς*_, *n*_*ς*_, and *p*_*ς*_ (*ς* = 1,2,…, *J* ^*σ*^) represent the variables *φ* of the *ς*th neuron of the first, second, and the *S*th layer, respectively. Finally, according to (12), the dynamics of the NN model (18) can be rewritten as the following LDI state-space representation:

(20) $$\begin{array}{c} \dot{X}\left(\;t\;\right)=\overset{\phi }{\underset{i=1}{\sum }}h_{i}\left(\;t\;\right)C_{i}\Lambda \left(\;t\;\right), \end{array}$$

where *h*_*i*_(*t*) ≥ 0, ∑_*i*=1_^*ϕ*^ *h*_*i*_(*t*) = 1, *ϕ* is a positive integer, and *C*_*i*_ is a constant matrix with appropriate dimension associated with *C*_*Ω*_^*σ*^. Moreover, the LDI state-space representation (20) can be rearranged as follows:

(21) $$\begin{array}{c} \dot{X}\left(\;t\;\right)=\overset{\phi }{\underset{i=1}{\sum }}h_{i}\left(\;t\;\right)\left\{\;A_{i}X\left(\;t\;\right)+\overset{g}{\underset{k=1}{\sum }}{\overset{-}{A}}_{ik}X\left(\;t-\tau_{k}\;\right)\;\right\}, \end{array}$$

where *A*_*i*_ and ${\overset{-}{A}}_{ik}$ are the partitions of *C*_*i*_ corresponding to the partitions of Λ^*T*^(*t*).

### 2.4. Fuzzy Controller

On the basis of the state-feedback control scheme, a model-based fuzzy controller is able to ensure that the slave system can synchronize with the master system. The output error is defined as $Y_{e}(t)\equiv \hat{Y}(t)-Y(t)$ and the fuzzy controller is in the following form:

(22) Control  Rule  l:  IF  e1t  is  Ml1  and  ⋯  and  eδt  is  MlδTHEN  Ut=−KlYet,

where *l* = 1,2,…, *m*, and *m* is the number of IF-THEN rules of the fuzzy controller, and *M*_*lη*_ (*η* = 1,2,…, *δ*) are the fuzzy sets. Therefore, the final output of this fuzzy controller can be inferred as follows:

(23) $$\begin{array}{c} U\left(\;t\;\right)=\frac{-\sum_{l=1}^{m}w_{l}\left(t\right)K_{l}Y_{e}\left(t\right)}{\sum_{l=1}^{m}w_{l}\left(t\right)}=-\overset{m}{\underset{l=1}{\sum }}{\overset{-}{h}}_{l}\left(\;t\;\right)K_{l}Y_{e}\left(\;t\;\right) \end{array}$$

with *w*_*l*_(*t*) ≡ ∏_*η*=1_^*δ*^ *M*_*lη*_(*e*_*η*_(*t*)), and *M*_*lη*_(*e*_*η*_(*t*)) is the grade of membership of *e*_*η*_(*t*) in *M*_*lη*_. Furthermore, ${\overset{-}{h}}_{l}(t)\equiv w_{l}(t)/\sum_{l=1}^{m}w_{l}(t)$ and $\sum_{l=1}^{m}{\overset{-}{h}}_{l}(t)=1$ for all *t*.

In the past, solving the feedback gains *K*_*l*_ (*l* = 1,2,…, *m*) was based on experience and the trial and error. It will therefore be advantageous to develop a powerful tool for solving suitable *K*_*l*_ (*l* = 1,2,…, *m*). Hence, the solving algorithm is constructed by GA in this paper [43].

From the above, the NN models of the master and slave chaotic systems are described by the following LDI state-space representation and a model-based fuzzy controller is designed by the state-feedback control scheme. Therefore, the master and slave chaotic systems can be rewritten as follows, respectively:

(24) Master:  X˙t=∑i=1ϕhitAiXt+∑k=1gA−ikXt−τk+BS∑l=1mwltKlιt,Yt=CXt+Sιt,

(25) Slave:  X^˙t=∑j=1ϕh^jtA^jX^t+∑k=1gA−^jkX^t−τk+BUt,Y^t=CX^t.

### 2.5. Improved Genetic Algorithm

To obtain a better performance of the proposed method, for the GA-based control gain design, this paper adopts the IGA whose superiority over standard GAs has been proposed and verified in [39, 43]. The key point of the IGA is that the chromosomes after crossover are averagely arranged in the central and boundary regions of the search domain. This crossover gives the next generation more potential to find the global optimal solution. The improved crossover is stated as follows [43, 52]:

(26) osc1os11os21⋯osno_vars1=P1+P22,

(27) osc2os12os22⋯osno_vars2=Pmax1−w+max⁡P1,P2w,

(28) osc3os13os23⋯osno_vars3=Pmin1−w+min⁡P1,P2w,

(29) osc4os14os24⋯osno_vars4=Pmax⁡+Pmin⁡1−w+P1+P2w2

in which

(30) Pmax=paramax1paramax2⋯paramaxno_vars,Pmin=paramin1paramin2⋯paraminno_vars

os_*c*_^1^ ~ os_*c*_^4^ are the chromosomes of the next generation, *P*_1_ and *P*_2_ are the two chromosomes chosen from the parent, and max(*P*_1_, *P*_2_) and min(*P*_1_, *P*_2_) are the new chromosomes in which the genes are the maximum and minimum, respectively, of the genes in the two chromosomes *P*_1_ and *P*_2_. *para*_max_^*ϑ*^, *para*_min_^*ϑ*^ are the upper bound and lower bound of the *ϑ*th genes, respectively, in the search space. The parameter *w* ∈ [0,1] is arbitrarily chosen. Equations (26) and (29) produce two new chromosomes distributed in the central region of the search domain, whereas (27) and (28) produce two new chromosomes distributed in the boundary region.

The fitness function for the application in this paper is defined as follows:

(31) $$\begin{array}{c} Fit\left(\;\Lambda \;\right)=\frac{1}{1+\sum_{t=0}^{t_{f}}\sum_{\eta =1}^{\delta }\left|e_{\eta }^{\Lambda }\left(t\right)\right|}+p\left(\;s_{\Lambda }\;\right) \end{array}$$

in which Fit(Λ) is the fitness value of the Λth chromosome in a population, *e*_*η*_^Λ^(*t*) is the error of the Λth chromosome in a population, and

(32) $$\begin{array}{c} p\left(\;s_{\Lambda }\;\right)=\left\{\begin{array}{cc} 0, & s_{\Lambda }<0\\ pv, & s_{\Lambda }\geq 0, \end{array}\right. \end{array}$$

where *s*_Λ_ is a variable for evaluating the stability of the systems and pv is a punishing value and will be set in the experiment.

The mutation operation is to change the genes of the chromosomes. Consequently, the features of the chromosomes inherited from their parents can be changed [43]. Three new offspring will be generated by the mutation operation:

(33) nosj=os1os2⋯osno_vars+b1Δnos1b2Δnos2⋯bno_varsΔnosno_vars,j=1,2,3,

where *b*_*i*_, *i* = 1, 2, 3,…, no_vars, can only take the value of 0 or 1; Δnos_*i*_, *i* = 1,2, 3,…, no_vars, are randomly generated numbers such that *para*_min_^*i*^ ≤ os_*i*_ + Δnos_*i*_ ≤ *para*_max_^*i*^. These three new offspring will then be evaluated using the fitness function of (31). A real number will be generated randomly and compared with a user-defined number $p_{a}\in \left[\begin{array}{cc} 0 & 1 \end{array}\right]$ . If the real number is smaller than *p*_*a*_, the one with the largest fitness value among the three new offspring will replace the chromosome with the smallest fitness *f*_*s*_ in the population. If the real number is larger than *p*_*a*_, the first offspring nos_1_ will replace the chromosome with the smallest fitness value *f*_*s*_ in the population if *f*(nos_1_) > *f*_*s*_; the second and the third offspring will do the same. *p*_*a*_ is effectively the probability of accepting a bad offspring in order to reduce the chance of converging to a local optimum.

## 3. Stability Analysis and Chaotic Synchronization via Fuzzy Control

In this section, the synchronization of multiple time-delay chaotic (MTDC) systems is examined under the influence of modeling error. The exponential synchronization scheme of the MTDC systems is described below.

### 3.1. Error Systems

From (1) and (2), the dynamics of the error system under the fuzzy control (5) can be described as follows:

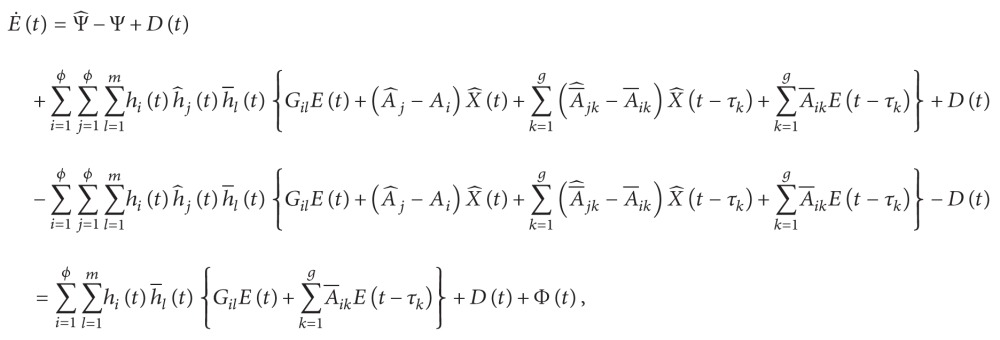

(34)
where

(35) Gil≡Ai−BCKl,Ψ^≡f^X^t+∑k=1gH^kX^t−τk+Ut,Ψ≡fXt+∑k=1gHkXt−τk

with


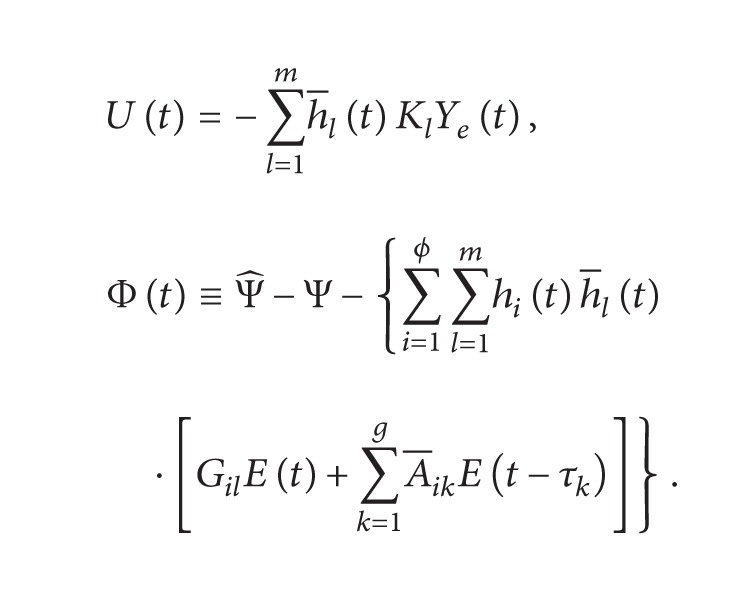

(36)
Suppose that there exists a bounding matrix Θ*R*_*il*_ such that

(37) $$\begin{array}{c} \left\|\;\Phi \left(\;t\;\right)\;\right\|\leq \left\|\;\overset{\phi }{\underset{i=1}{\sum }}\;\overset{m}{\underset{l=1}{\sum }}h_{i}\left(\;t\;\right){\overset{-}{h}}_{l}\left(\;t\;\right)\Theta R_{il}E\left(\;t\;\right)\;\right\| \end{array}$$

for the trajectory *E*(*t*), and the bounding matrix Θ*R*_*il*_ can be described as follows:

(38) $$\begin{array}{c} \Theta R_{il}=\varepsilon_{il}R, \end{array}$$

where *R* is the specified structured bounding matrix and ‖*ε*_*il*_‖ ≤ 1, for *i* = 1,2,…, *ϕ*; *l* = 1,2,…, *m*. Equations (37) and (38) show that

(39) ΦTtΦt≤∑i=1ϕ ∑l=1mhith−ltREtεil·∑i=1ϕ ∑l=1mhith−ltεilREt≤REtT·REt.

Namely, Φ(*t*) is bounded by the specified structured bounding matrix *R*.

**Remark 1 (see [35]).**

The following simple example describes the procedures for determining *ε*_*il*_ and *R*. First, assume that the possible bounds for all elements in Θ*R*_*il*_ are

(40) $$\begin{array}{c} \Theta R_{il}=\left[\begin{array}{ccc} \Theta r_{il}^{11} & \Theta r_{il}^{12} & \Theta r_{il}^{13}\\ \Theta r_{il}^{21} & \Theta r_{il}^{22} & \Theta r_{il}^{23}\\ \Theta r_{il}^{31} & \Theta r_{il}^{32} & \Theta r_{il}^{33} \end{array}\right], \end{array}$$

where −*r* ^*qs*^ ≤ Θ*r*_*il*_^*qs*^ ≤ *r* ^*qs*^ for some *r*_*il*_^*qs*^ with *q*, *s* = 1,2, 3; *i* = 1,2,…, *ϕ*, and *l* = 1,2,…, *m*.

A possible description for the bounding matrix Θ*R*_*il*_ is

(41) $$\begin{array}{c} \Theta R_{il}=\left[\begin{array}{ccc} \varepsilon_{il}^{11} & 0 & 0\\ 0 & \varepsilon_{il}^{22} & 0\\ 0 & 0 & \varepsilon_{il}^{33} \end{array}\right]\left[\begin{array}{ccc} r_{}^{11} & r_{}^{12} & r_{}^{13}\\ r_{}^{21} & r_{}^{22} & r_{}^{23}\\ r_{}^{31} & r_{}^{32} & r_{}^{33} \end{array}\right]=\varepsilon_{il}R, \end{array}$$

where −1 ≤ *ε*_*il*_^*qq*^ ≤ 1 for *q* = 1,2, 3. Notice that *ε*_*il*_ can be chosen by other forms as long as ‖*ε*_*il*_‖ ≤ 1. The validity of (37) is then checked in the simulation. If it is not satisfied, we can expand the bounds for all elements in Θ*R*_*il*_ and repeat the design procedure until (37) holds.

### 3.2. Delay-Dependent Stability Criterion for Exponential *H* ^*∞*^ Synchronization

In this subsection, a delay-dependent criterion is proposed to guarantee the exponential stability of the error system described in (34). Moreover, in general, there will always be some noise or disturbances that may cause instability. The effect of the external disturbance *D*(*t*) will negatively affect the performance of chaotic systems. To reduce the effect of the external disturbance, an optimal *H* ^*∞*^ scheme is used to design a fuzzy control such that the effect of the external disturbance on control performance can be attenuated to a minimum level. In other words, the fuzzy controller (5) simultaneously realizes exponential synchronization and achieves the optimal *H* ^*∞*^ control performance in this study.

Before examining the stability of the error system, some definitions and lemma are given below.

**Lemma 2 (see [53]).**

For the real matrices *A* and *B* with appropriate dimension,

(42) $$\begin{array}{c} A^{T}B+B^{T}A\leq ƛA^{T}A+{ƛ}^{-1}B^{T}B, \end{array}$$

where *ƛ* is a positive constant.

**Definition 3 (see [54]).**

The slave system (2) can exponentially synchronize with the master system (1) (i.e., the error system (38) is exponentially stable) if there exist two positive numbers *α* and *β* so that the synchronization error satisfies

(43) $$\begin{array}{c} \left\|\;E\left(\;t\;\right)\;\right\|\leq \alpha \exp\left(\;-\beta \left(\;t-t_{0}\;\right)\;\right),\;\forall t\geq 0, \end{array}$$

where the positive number *β* is called the exponential convergence rate.

**Definition 4 (see [13–16]).**

The master system (1) and slave system (2) are said to be in exponential *H* ^*∞*^ synchronization if the following conditions are satisfied:

(i) With zero disturbance (i.e., *D*(*t*) = 0), the error system (34) with the fuzzy controller (5) is exponentially stable.
(ii) Under the zero initial conditions (i.e., *E*(*t*) = 0 for *t* ∈ [−*τ*_max_, 0], in which *τ*_max_ is the maximal value of *τ*_*k*_'s) and a given constant *ρ* > 0, the following condition holds:
(44) ΘYet,Dt∫0∞YeTtYetdt−ρ2∫0∞DTtDtdt≤0,

where the parameter *ρ* is called the *H* ^*∞*^-norm bound or the disturbance attenuation level. If the minimum *ρ* is found to satisfy the above conditions (i.e., the error system can reject the external disturbance as strongly as possible), the fuzzy controller (5) is an optimal *H* ^*∞*^ synchronizer [14].

**Theorem 5 .**

For given positive constants *a* and *n*, if there exist two symmetric positive definite matrices *P* and *ψ*_*k*_, as well as two positive constants *ξ* and *ρ*, so that the following inequalities hold, then the exponential *H* ^*∞*^ synchronization with the disturbance attenuation *ρ* is guaranteed via the fuzzy controller (5):

(45a) Δil∑k=1gτkPGil+∑k=1gτkGilTP+∑k=1gψk+ngRTR+CTC+I+∑k=1gτk2P2ξ−1+n−1+ga−1<0,

(45b) ∇ikgaA−ikTA−ik−ψk<0,

(45c) ρξg,

where *G*_*il*_ ≡ *A*_*i*_ − *BCK*_*l*_, for *i* = 1,2,…, *ϕ*; *k* = 1,2,…, *g*, and *l* = 1,2,…, *m*.

**Proof**

Let the Lyapunov function for the error system (34) be defined as

(46) Vt=∑k=1gETtτkPEt+∑k=1g∫0τkETt−πψkEt−πdπ,

where the weighting matrices *P* = *P* ^*T*^ > 0 and *ψ*_*k*_ = *ψ*_*k*_^*T*^ > 0. We then evaluate the time derivative of *V*(*t*) on the trajectories of (34) to obtain

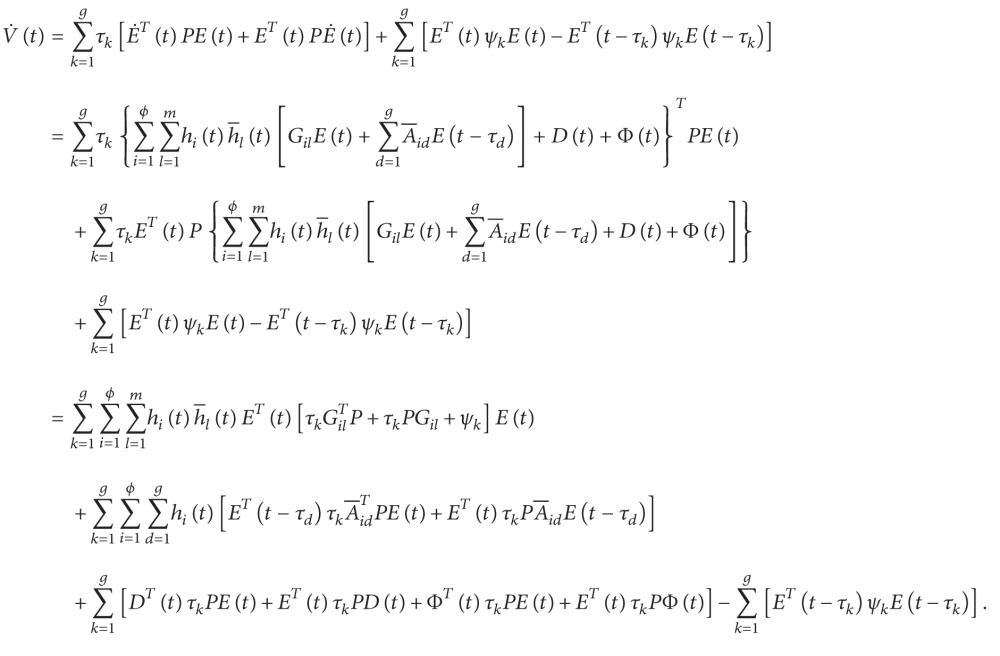

(47)
According to Lemma 2 and (47), we have

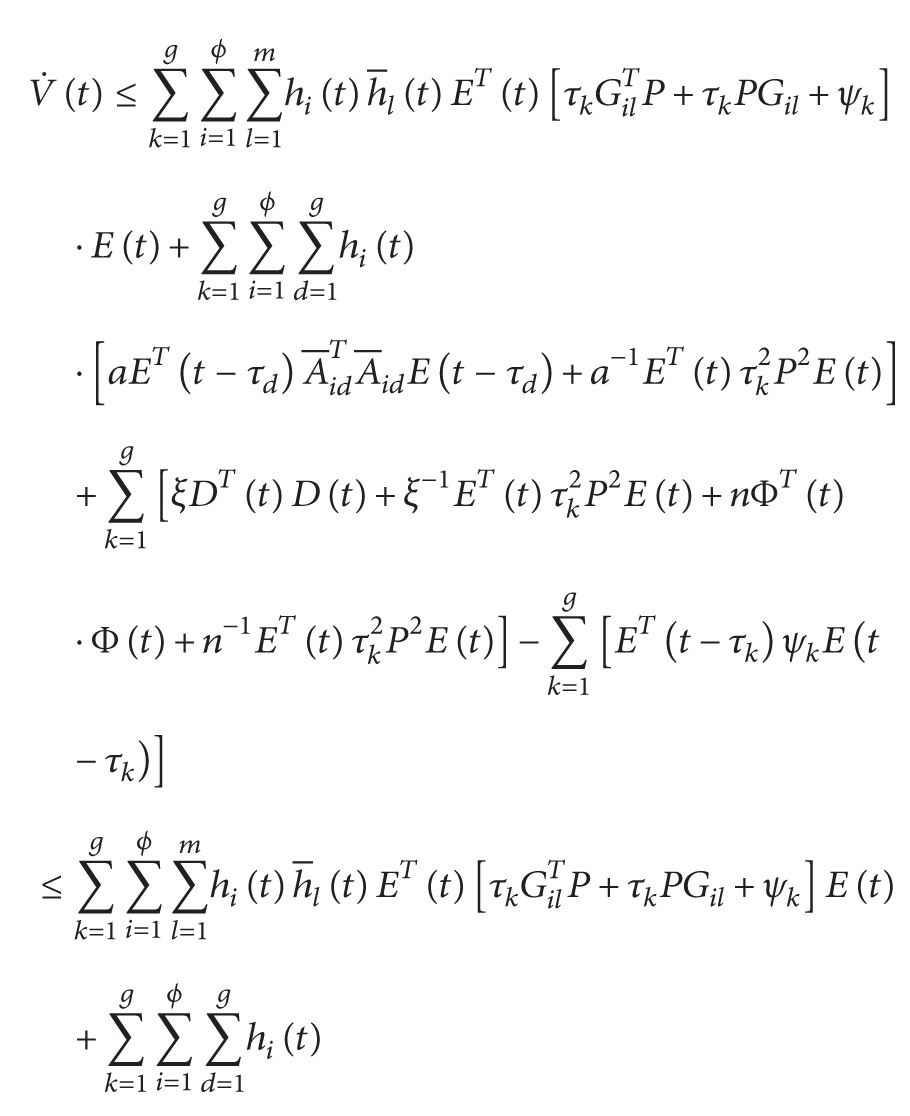

(48)



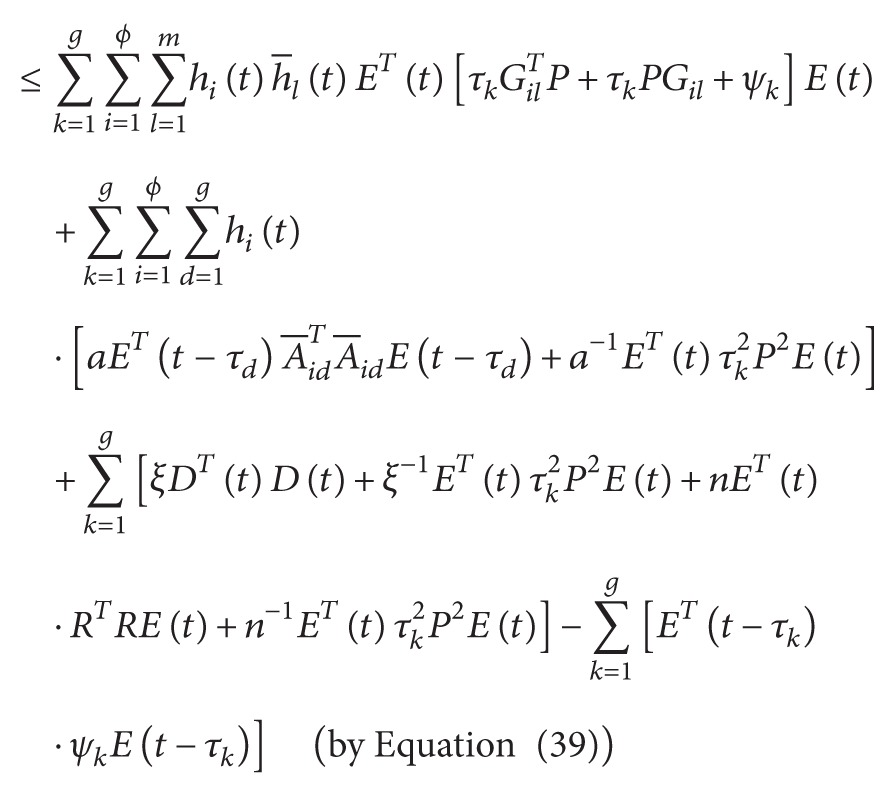

(49)



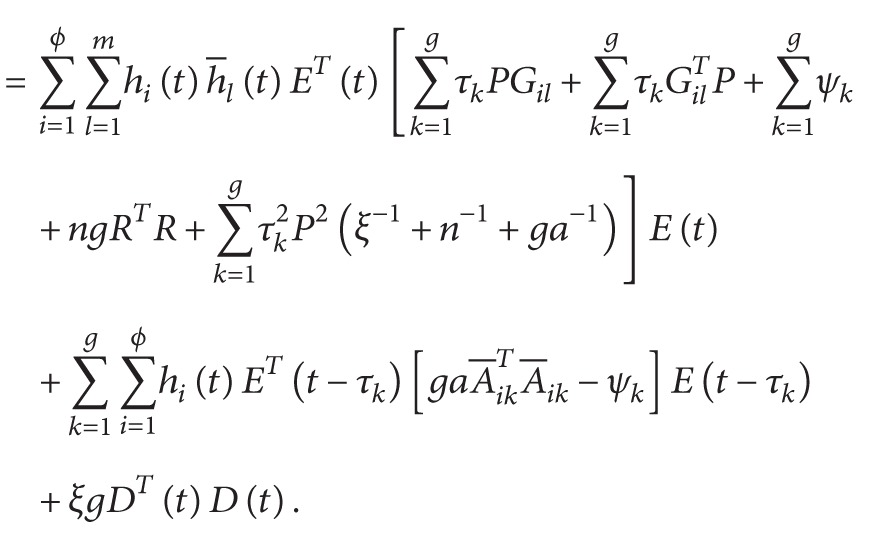

(50)
From (50), we have

(51) V˙t+YeTtYet−ρ2DTtDt≤∑i=1ϕ ∑l=1mhith−ltETtΔilEt+∑i=1ϕ ∑k=1ghitETt−τk∇ikEt−τk+ξg−ρ2DTtDt≤∑i=1ϕ ∑l=1mhith−ltλmax⁡ΔilETtEt+∑i=1ϕ ∑k=1ghitλmax⁡∇ikETt−τkEt−τk+ξg−ρ2DTtDt<0,

where

(52) Δil≡∑k=1gτkPGil+∑k=1gτkGilTP+∑k=1gψk+ngRTR+CTC+I+∑k=1gτk2P2ξ−1+n−1+ga−1see 45a∇ik≡gaA−ikTA−ik−ψksee 45b.

Integrating (51) from *t* = 0 to *t* = *∞*, the following inequality is obtained:

(53) V∞−V0+∫0∞YeTtYetdt−ρ2∫0∞DTtDtdt≤0.

With zero initial conditions, (i.e., *E*(*t*) ≡ 0 for *t* ∈ [−*τ*_max_, 0]), we have

(54) $$\begin{array}{c} \overset{\infty }{\underset{0}{\int }}Y_{e}^{T}\left(\;t\;\right)Y_{e}\left(\;t\;\right)dt\leq \rho^{2}\overset{\infty }{\underset{0}{\int }}D^{T}\left(\;t\;\right)D\left(\;t\;\right)dt. \end{array}$$

That is (44) and the *H* ^*∞*^ control performance is achieved with a prescribed attenuation *ρ*.

Since

(55) ∑k=1gτkλminPETtEt≤∑k=1gτkETtPEt=Vt−∑k=1g∫0τkETt−πψkEt−πdπ<Vtfrom 46,

we can get the following inequality from (51):

(56) V˙t+YeTtYet−ρ2DTtDt<∑i=1ϕ ∑l=1mhith−ltλmax⁡Δil∑k=1gτkλmin⁡PVt<0.

Then, we can obtain

(57) $$\begin{array}{c} {\left.\;V\left(\;t\;\right)\;\right|}_{D\left(t\right)=0}\leq V\left(\;t_{0}\;\right)\exp\overset{-}{\beta }\left(\;t-t_{0}\;\right), \end{array}$$

where $\overset{-}{\beta }=\sum_{i=1}^{\phi }\sum_{l=1}^{m}h_{i}(t){\overset{-}{h}}_{l}(t)\left[\lambda_{max}(\Delta_{il})/\sum_{k=1}^{g}\tau_{k}\lambda_{min}\left(P\right)\right]$ .

Equations (46) and (57) show that

(58) ∑k=1gτkλmin⁡PETtEt≤∑k=1gETtτkPEt<Vt0exp⁡β−t−t0−∑k=1g∫0τkETt−πψkEt−πdπ<Vt0exp⁡β−t−t0.

That is, ${\left\|E(t)\right\|}^{2}\leq \left(V\left(t_{0}\right)/\sum_{k=1}^{g}\tau_{k}\lambda_{min}\left(P\right)\right)\exp\overset{-}{\beta }(t-t_{0})$ . Therefore, we conclude that

(59) Et≤αexp⁡−βt−t0with  α≡Vt0∑k=1gτkλmin⁡P>0,  β≡−12β−>0.

Hence, on the basis of Definition 3, the error system (34) with the fuzzy controller (5) is exponentially stable for *D*(*t*) = 0.

**Corollary 6 .**

Equations (45a) and (45b) can be reformulated into LMIs via the following procedure.

By introducing the new variables, *Q* = *P* ^−1^, *F*_*l*_ = *K*_*l*_ *Q* and ${\overset{-}{\psi }}_{k}=Q\psi_{k}Q$ , (45a) and (45b) can be rewritten as follows:

(60a) ∑k=1gτkAiQ−BFl+QAiT−FlTBT+∑k=1gψ−k+ngQRTRQ+QCTCQ+QIQ+∑k=1gτk2ξ−1+n−1+ga−1I<0,

(60b) gaQA−ikTA−ikQ−ψ−k<0

for *i* = 1,2,…, *ϕ*; *k* = 1,2,…, *g*, and *l* = 1,2,…, *m*. According to Schur's complement [26], it is easy to show that the linear matrix inequalities in (60a) and (60b) are equivalent to the following LMIs in (61a) and (61b):

(61a) ΞQRTQRQT−ng−1I0Q0−I<0,

(61b) −ψ−kQA−ikTA−ikQ−ga−1I<0,

where

(62) Ξ≡∑k=1gτkAiQ−∑k=1gτkBFl+∑k=1gτkQAiT−∑k=1gτkFlTBT+∑k=1gψ−k+∑k=1gτk2ξ−1+n−1+ga−1I+QCTCQ.

Thus, Theorem 5 can be transformed into an LMI problem, and efficient interior-point algorithms are now available in Matlab LMI Solver to solve this problem.

**Corollary 7 (see [55]).**

In order to verify the feasibility of solving the inequalities in (61a) and (61b) using the LMI Solver (Matlab), interior-point optimization techniques are utilized to compute feasible solutions. These techniques require that the LMI systems are constrained to be strictly feasible; that is, the feasible set has a nonempty interior. For feasibility problems, the LMI Solver by* feasp* (*feasp *is the syntax used to test the feasibility of a system of LMIs in MATLAB) is shown as follows:

(63a) $$\begin{array}{c} Find\;x\;such\;that\;the\;LMI\;L\left(\;x\;\right)<0 \end{array}$$

(in this study, (63a) can be represented as (61a) and (61b)) as

(63b) $$\begin{array}{c} Minimize\;\;t\;\;subject\;\;to\;\;L\left(\;x\;\right)<t\times I, \end{array}$$

where *L*(*x*) is symmetric matrix and *I* is identity matrix.

From the above, the LMI constraint is always strictly feasible in *x*, *t* and the original LMI (63a) is feasible if and only if the global minimum *t*_min_ (the global minimum *t*_min_ is the scalar value returned as the output argument by* feasp*) of (63b) satisfies *t*_min_ < 0. In other words, if *t*_min_ < 0 will satisfy (61a) and (61b), then the stability conditions (45a) and (45b) in Theorem 5 can be met. The obtained fuzzy controller (5) can then exponentially stabilize the error system, and the *H* ^*∞*^ control performance is achieved at the same time.

**Corollary 8 .**

In order to achieve exponential optimal *H* ^*∞*^ synchronization, the fuzzy control design is formulated as the following constrained optimization problem:

(64) minimize ρ>ξgsubject  to Q=QT>0,  ψ−k=ψ−kT>0,61a  and  61b.

More details on searching for the minimum *ρ* are given as follows: the positive constant *ξ* is minimized by the mincx function of Matlab LMI Toolbox. Therefore, the minimum disturbance attenuation level $\rho_{min}>\sqrt{\xi_{min}g}$ can be obtained.

**Remark 9 .**

In order to reduce the computational burden, this study sets the positive constants *a* and *n* as unity.

**Remark 10 .**

It is important to reduce the effect of external disturbances in the synchronization process. The *H* ^*∞*^-norm bound *ρ* is generally chosen as a positive small value less than unity for the attenuation of the disturbance. A smaller *ρ* is desirable as this yields better performance. However, a smaller *ρ* will result in a smaller *ξ*, making stability condition (45a) more difficult to satisfy.

**Remark 11 .**

According to (37), Φ(*t*) is assumed to be bounded by the specified structured bounding matrix *R*, and a larger Φ(*t*) results in a larger *R*. Since the matrices Δ_*il*_ must be negative definite to meet stability condition (45a), a larger *R* will make Theorem 5 more difficult to satisfy.

**Remark 12 .**

Since inequality (60a) must be negative definite to meet the stability condition, the larger delay *τ*_*k*_ will make Theorem 5 more difficult to satisfy.

## 4. Algorithm

The complete design procedure can be summarized as follows.

**Problem 1 .**

Given two multiple time-delay chaotic systems with different initial conditions and cryptography, the problem is centered on how to synthesize a fuzzy controller to realize the exponential optimal *H* ^*∞*^ synchronization and achieve a more secure communication system.

We can solve this problem based on the following steps.

*Step 1*. The input message (plaintext) and encryption key form an encrypted signal via an *n*-shift cipher.

*Step 2*. The encrypted message is then combined in the master system.

*Step 3*. Construct the neural network (NN) models of the master system (6) and the slave system (7), respectively. On the basis of the interpolation method, the NN models are then converted into LDI state-space representations.

*Step 4*. On the basis of the state-feedback control scheme, the feedback gains of the model-based fuzzy controller (23) are synthesized to exponentially stabilize the error system by the Matlab LMI Toolbox.

*Step 5*. Based on IGA process shown in Section 2.5, obtain the feedback gains to stabilize the MTDC systems.

*Step 6*. Define the synchronization error $E(t)=\hat{X}(t)-X(t)$ , and the dynamics of the error system (34) can then be obtained.

*Step 7*. Based on Corollary 8, the positive constant *ξ* is minimized by the mincx function of Matlab LMI Toolbox; we then have the minimum disturbance attenuation level.

*Step 8*. The matrices *Q*, *F*_*l*_, and ${\overset{-}{\psi }}_{k}$ can be obtained with the minimum disturbance attenuation *ρ*_min_.

*Step 9*. Based on the decryption function, we can then retrieve the original message from the encryption signal.

## 5. Numerical Example

The following example is given to illustrate the effectiveness of the proposed algorithm.

**Problem 2 .**

**Figure 4:**
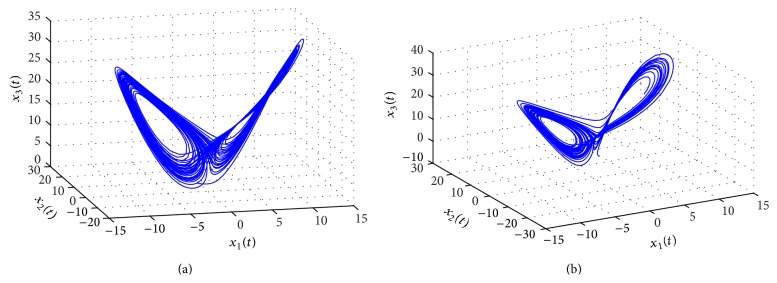
(a) Chaotic behavior of the master system (65). (b) Chaotic behavior of the slave system (66) without control.

The purpose of this example is to synthesize a fuzzy controller and cryptography to achieve optimal *H* ^*∞*^ exponential synchronization and a more secure communication system. Consider a pair of modified multiple time-delay Chen's chaotic systems in master-slave configuration, described as follows (Figure 4):

(65) x˙1t=5x2t−x1t,x˙2t=20x1t−x2t−0.15−x1tx3t,x˙3t=x1tx2t−83x3t−0.01,yt=x1t,

(66) x^˙1t=5x^2t−x^1t+Dt+u1t,x^˙2t=20x^1t−x^2t−0.15−x^1tx^3t+Dt+u2t,x^˙3t=x^1tx^2t−83x^3t−0.01+Dt+u3t,yt=x^1t,

where [*x*_1_(*t*) *x*_2_(*t*) *x*_3_(*t*)]^*T*^ and ${\left[{\hat{x}}_{1}\left(t\right)\;{\hat{x}}_{2}\left(t\right)\;{\hat{x}}_{3}\left(t\right)\right]}^{T}$ are the state vectors of master and slave systems, respectively. Let the different initial conditions of master and slave systems be [*x*_1_(0) = −0.7 *x*_2_(0) = 4 *x*_3_(0) = 1] and $\left[{\hat{x}}_{1}(0)=1.1\;{\hat{x}}_{2}(0)=-1\;{\hat{x}}_{3}(0)=-1.2\right]$ , respectively, and let the external disturbance be *D*(*t*) = 0.1sin⁡(1.3*t*).

**Figure 5:**
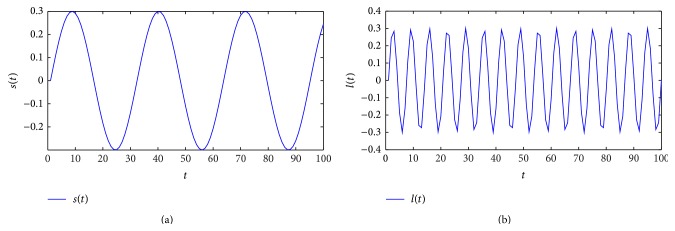
(a) Original signal *s*(*t*). (b) Encrypted signal *ι*(*t*).

*Solution.*  We can solve the above problem according to the following steps (Figure 5).

*Step 1*. Assuming a 6-shift cipher, the ciphertext is defined by

(67) ιtζst=FFF⋯︸6st,⋯ϑt,ϑt,ϑt︸6,

where *s*(*t*) = 0.3sin⁡(2*t*) is the input message, *ϑ*_  _(*t*) = 6 is the encryption key, and

(68) Fst,ϑt=st+ϑt+1,−1≤st+ϑt≤−0.5st+ϑt,−0.5<st+ϑt<0.5st+ϑt−1,0.5≤st+ϑt≤1.

*Step 2*. The encrypted message is then combined in the master system (65):

(69) x˙1t=5x2t−x1t+ιt,x˙2t=20x1t−x2t−0.15−x1tx3t,x˙3t=x1tx2t−83x3t−0.01,yt=x1t+ιt.

**Figure 6:**
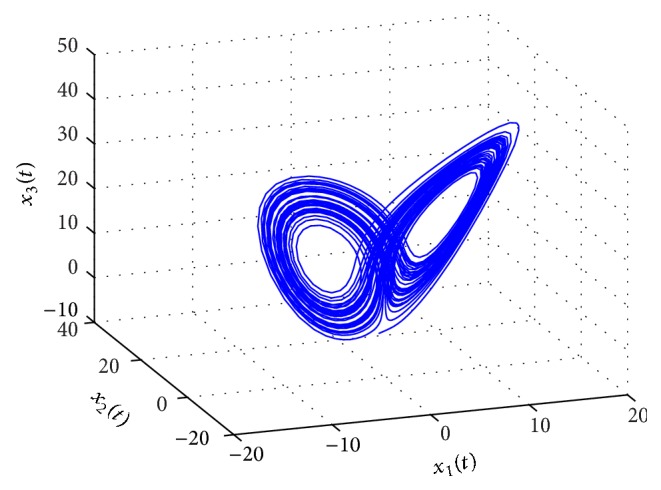
Chaotic behavior of the master system (69).

Figure 6 shows the chaotic behaviors of the master system (69) with encrypted signal.

*Step 3*. Establish the NN models for master and slave systems via backpropagation algorithm, respectively. First, the NN model to approximate the master chaotic system is constructed by 10-3, and the transfer functions of all hidden neurons are chosen as follows:

(70) Tvςσt=21+exp⁡−vςσt/0.5−1,for  σ=1.

On the other hand, the transfer functions of all output neurons are chosen as follows:

(71) $$\begin{array}{c} T\left(\;v_{\varsigma }^{\sigma }\left(\;t\;\right)\;\right)=v_{\varsigma }^{\sigma }\left(\;t\;\right),\;for\;\;\sigma =2. \end{array}$$

After training, we can obtain the following connection weights (the indices in *W*_*ςϑ*_^*σ*^ state that the weight of the *σ*th layer in the NN model represents the connection to the *ς*th neuron from the *ϑ*th source): 

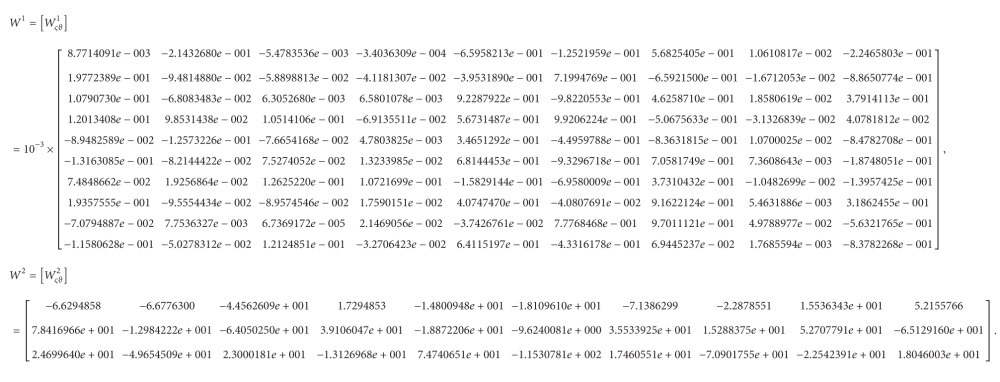

(72)
Then, the net inputs of the *σ*th (*σ* = 1,2) layer are (the symbol *v*_*ς*_^*σ*^ denotes the net input of the *ς*th neuron of the *σ*th layer in the NN model, and the indices *σ* and *ς* shown in *h*_*ςφ*_^*σ*^ (*φ* = 1,2) indicate the same thing) as follows:

(73a) vς1tWς11x1t+Wς21x2t+Wς31x3t+Wς41x1t−0.15+Wς51·0+Wς61·0+Wς71·0+Wς81x2t−0.01+Wς91·0,ς=1,2,3,4,5,6,7,8,9,10,

(73b) vς2tWς12Tv11t+Wς22Tv21t+Wς32Tv31t+Wς42Tv41t+Wς52Tv51t+Wς62Tv61t+Wς72Tv71t,ς=1,2,3,

(74) X˙tx˙1tx˙2tx˙3t=Tv13tTv23tTv33t.

According to (14), the minimum and the maximum of the derivative of each transfer function shown in (70) and (71) can be obtained as follows:

(75) gς010,gς021,gς11gς12=1,for  ς=1,2,…,Jσ.

To simplify the notation, we let *g*_*ς*0_^1^ = *g*_0_^1^, *g*_*ς*1_^1^ = *g*_1_^1^, *g*_*ς*0_^2^ = *g*_0_^2^, and *g*_*ς*1_^2^ = *g*_1_^2^. Then, based on the interpolation method, we have 

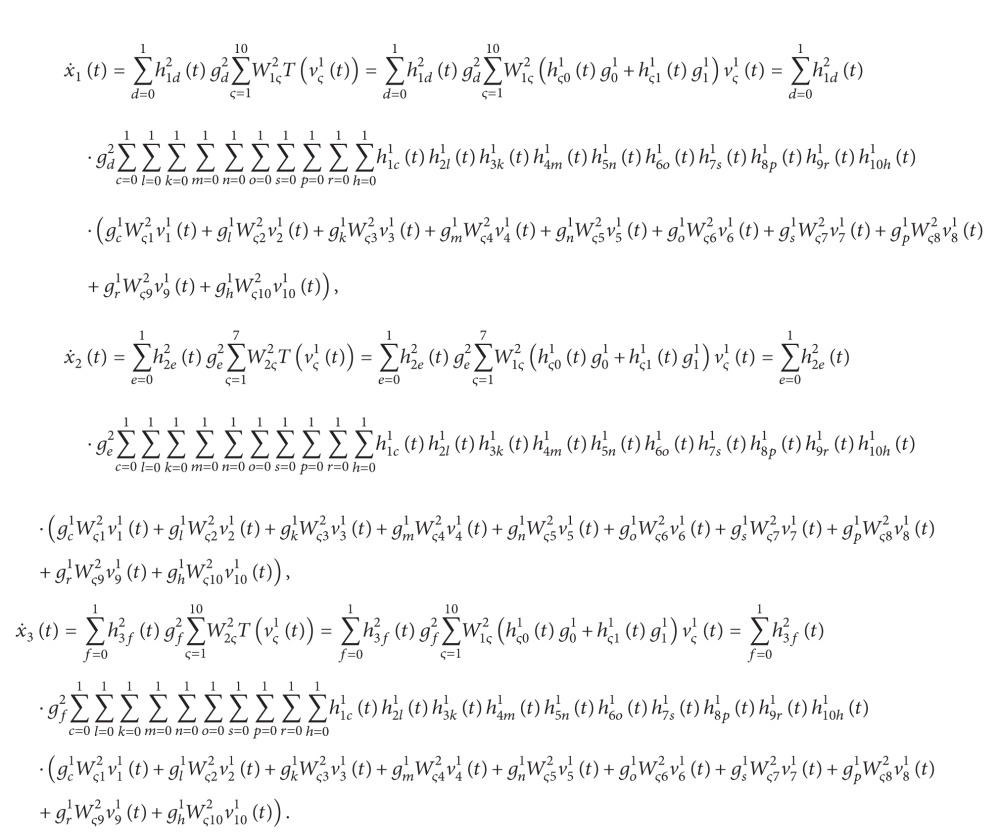

(76)
Based on (15), let

(77) G1=gc10000000000gl10000000000gk10000000000gm10000000000gn10000000000go10000000000gs10000000000gp10000000000gr10000000000gh1,G2=gd2000ge2000gf2

and then,

(78) Edefclkmnosprh≡G2W2G2W1=ΥRℵ3×9,R=1,2,3;  ℵ=1,2,…,9.

Plugging (73a)–(73b) into (76) leads to

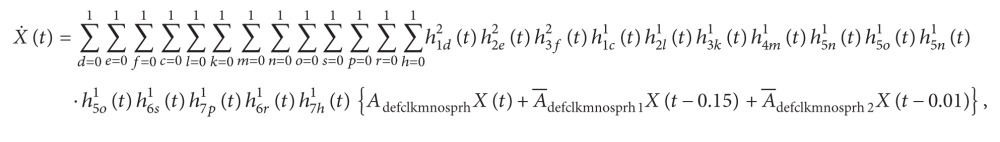

(79)
where

(80) Adefclkmnosprh=Υ11Υ12Υ13Υ21Υ22Υ23Υ31Υ32Υ33,A−defclkmnosprh 1=Υ14Υ15Υ16Υ24Υ25Υ26Υ34Υ35Υ36,A−defclkmnosprh 2=Υ17Υ18Υ19Υ27Υ28Υ29Υ37Υ38Υ39,A−defclkmnosprh 3=Υ110Υ111Υ112Υ210Υ211Υ212Υ310Υ311Υ312,Xt=x1tx2tx3tT,Xt−0.15=x1t−0.1500T,Xt−0.01=0x2t−0.010T.

Next, by renumbering the matrices shown in (79), the NN model of the master system can be rewritten as the following LDI state-space representation:

(81) X˙t=∑i=18192hitAiXt+∑k=12A−ikXt−τk+∑l=12h−ltKlιt,

where *τ*_1_ = 0.15, *τ*_2_ = 0.01,

(82) $$\begin{array}{c} A_{1}=A_{0000000000000},\\ \vdots \\ A_{8191}=A_{111111111110},\\ A_{8192}=A_{111111111111}, \end{array}$$

(83) $$\begin{array}{c} {\overset{-}{A}}_{11}=A_{00000000000001},\\ \vdots \\ {\overset{-}{A}}_{81911}=A_{1111111111101},\\ {\overset{-}{A}}_{81921}=A_{1111111111111},\\ \vdots \\ {\overset{-}{A}}_{81912}=A_{1111111111102},\\ {\overset{-}{A}}_{81922}=A_{1111111111112}. \end{array}$$

(84) $$\begin{array}{c} {\overset{-}{A}}_{12}=A_{00000000000002},\\ \vdots \\ {\overset{-}{A}}_{81912}=A_{1111111111102},\\ {\overset{-}{A}}_{81922}=A_{1111111111112}. \end{array}$$

Similarly, the connection weights of the NN model for the slave system are obtained as follows:

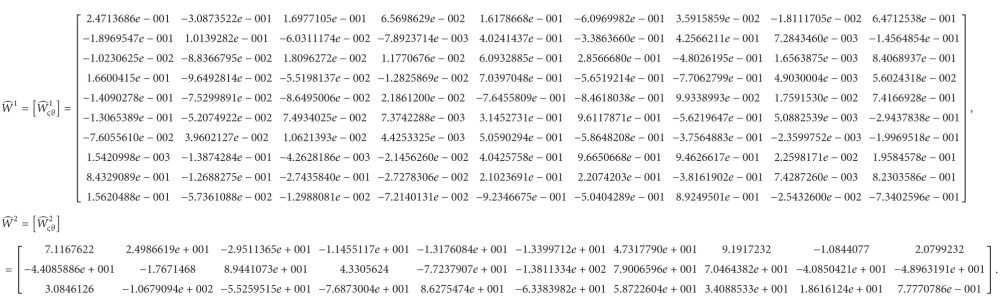

(85)



*Step 4*. The procedures of constructing the NN model for the slave system are similar to those of for that the master system, and we then have the NN model of the slave system

**Figure 7:**
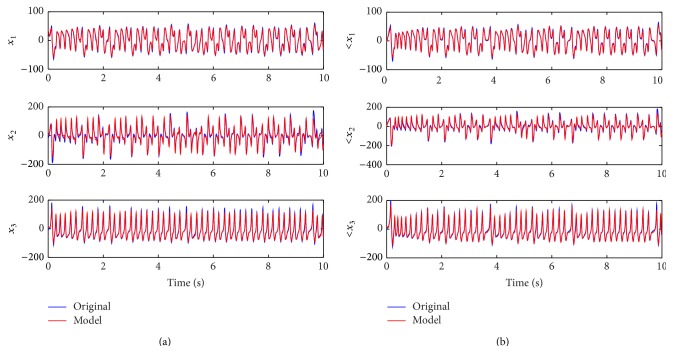
(a) The responses of $\dot{X}(t)$ for original system and NN model. (b) The responses of $\dot{\hat{X}}(t)$ for original system and NN model.

(86) X^˙t=∑j=18192h^jtA^jX^t+∑k=12A−^jkX^t−τk+BUt

with *τ*_1_ = 0.15, *τ*_2_ = 0.01, and *B* is a identity matrix. The responses of $\dot{X}(t)$ and $\dot{\hat{X}}(t)$ for the original systems and the NN models are shown in Figures 7(a) and 7(b). 

**Figure 8:**
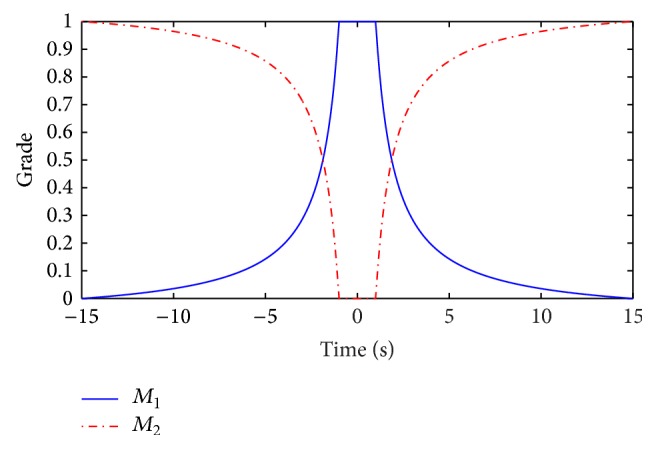
Membership functions of the fuzzy controller.

*Step 5*. To synchronize the master and slave systems, a fuzzy controller is synthesized as follows:

(87) Control  Rule  1:  IF  e1t  is  M1,  THEN  Ut=−K1Yet,Control  Rule  2:  IF  e1t  is  M2,  THEN  Ut=−K2Yet,

where *M*_1_ and *M*_2_ are the membership functions for each *e*_1_ (see Figure 8):

(88a) M1e1=−e1/15+114e1/15,e1≥11,−1<e1<1,−e1/15−114e1/15,e1≤−1,

(88b) M2e1=1−M1e1.

According to (18), we have the overall fuzzy controller:

(89) $$\begin{array}{c} U\left(\;t\;\right)=-\frac{\sum_{l=1}^{2}w_{l}\left(t\right)K_{l}Y_{e}\left(t\right)}{\sum_{l=1}^{2}w_{l}\left(t\right)}=-\overset{2}{\underset{l=1}{\sum }}{\overset{-}{h}}_{l}\left(\;t\;\right)K_{l}Y_{e}\left(\;t\;\right) \end{array}$$

with *w*_*l*_(*t*) ≡ *M*_*l*_(*e*_1_(*t*)), ${\overset{-}{h}}_{l}(t)\equiv w_{l}(t)/\sum_{l=1}^{2}w_{l}(t)$ .

According to (34), the dynamics of the error system is obtained as follows:

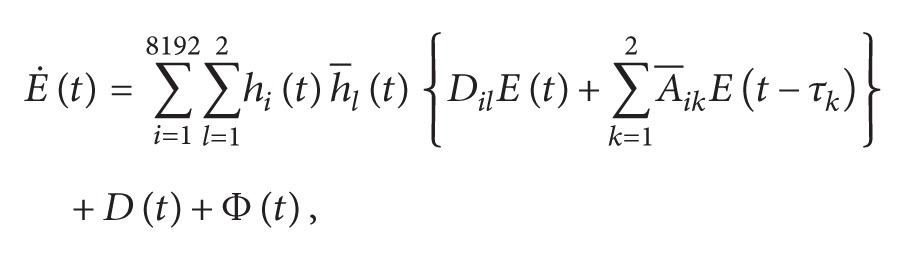

(90)
where *D*_*il*_ ≡ *A*_*i*_ − *BCK*_*l*_, $\hat{\Gamma }\equiv f(\hat{X}(t))+\sum_{k=1}^{2}H_{k}(\hat{X}(t-\tau_{k}))+U(t)$ with *U*(*t*) = −∑_*l*=1_^2^ *h*_*l*_(*t*)*K*_*l*_ *Y*_*e*_(*t*), Γ ≡ *f*(*X*(*t*)) + ∑_*k*=1_^2^ *H*_*k*_(*X*(*t* − *τ*_*k*_)), $\Phi (t)\equiv \hat{\Gamma }-\Gamma -\left\{\sum_{i=1}^{8192}\sum_{l=1}^{2}h_{i}(t){\overset{-}{h}}_{l}(t)[D_{il}E(t)+\sum_{k=1}^{2}{\overset{-}{A}}_{ik}E(t-\tau_{k})]\right\}$ .

*Step 6*. Design the feedback gains. Based on IGA, obtain the performance of the feedback gains in this paper.

The Improved GA (IGA) is adopted for its better performance over traditional GA [39, 43]. Before executing the search process of the IGA, some specifications are given in Table 1. Note that parameters *w* and *P*_*m*_ are determined by repeating the experiments with various *w* and *P*_*m*_ as shown in Table 2. The values of *w* and *P*_*m*_ with best fitness value are selected.

After executing the IGA search process, the resulting feedback gains are obtained as follows:

(91a) K1=103×9.4701−0.0034−0.00180.00349.47010.00020.0018−0.00029.4701,

(91b) K2=103×9.47010.00060.0003−0.00069.47010.0000−0.00030.00009.4701.

**Figure 9:**
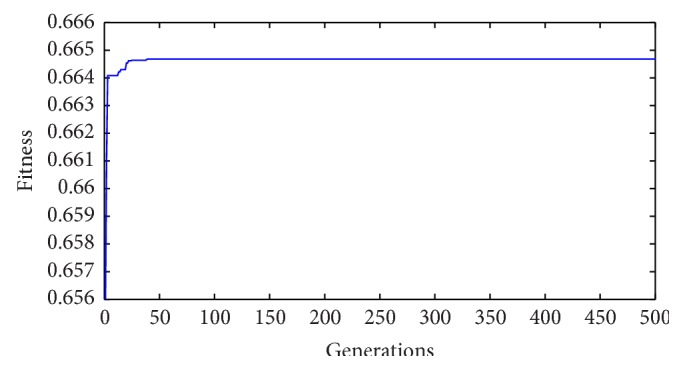
Fitness value of IGA.

Evolution of the fitness values are shown in Figure 9.

*Step 7*. Based on (75) and (79)–(90), the LMI in (61a) and (61b) can be solved via Matlab LMI Toolbox. In accordance with Remark 1, the specified structured bounding matrices *Y* and *κ*_*il*_ are set to be $Y=\left[\begin{array}{ccc} 12000 & 0 & 0\\ 0 & 12000 & 0\\ 0 & 0 & 12000 \end{array}\right]$ and $\kappa_{il}=\left[\begin{array}{ccc} 1 & 0 & 0\\ 0 & 1 & 0\\ 0 & 0 & 1 \end{array}\right]$ . Based on Corollary 8, the positive constant *c* is minimized by the mincx function of Matlab LMI Toolbox: *ξ*_min_ = 1.315 × 10^−2^; we then have the minimum disturbance attenuation level *ρ*_min_ = 0.11467.

*Step 8*. The common solutions, *P*, *F*_1_, *F*_2_, ${\overset{-}{\psi }}_{1}$ , and ${\overset{-}{\psi }}_{2}$ , of stability conditions (45b) and (45c) can be obtained with the best value *t*_min_ of LMI Solver (Matlab) as −8.68953 × 10^−7^:

(92) P105×5.46470.00010.00000.00015.4647−0.00020.0000−0.00025.4647,

(93) F10.01330.00000.00000.00000.01330.00000.00000.00000.0133,

(94) F20.01330.00000.00000.00000.01330.00000.00000.00000.0133,

(95) ψ−1ψ−2=2.01080.00000.00000.00002.01080.00000.00000.00002.0108.

**Figure 10:**
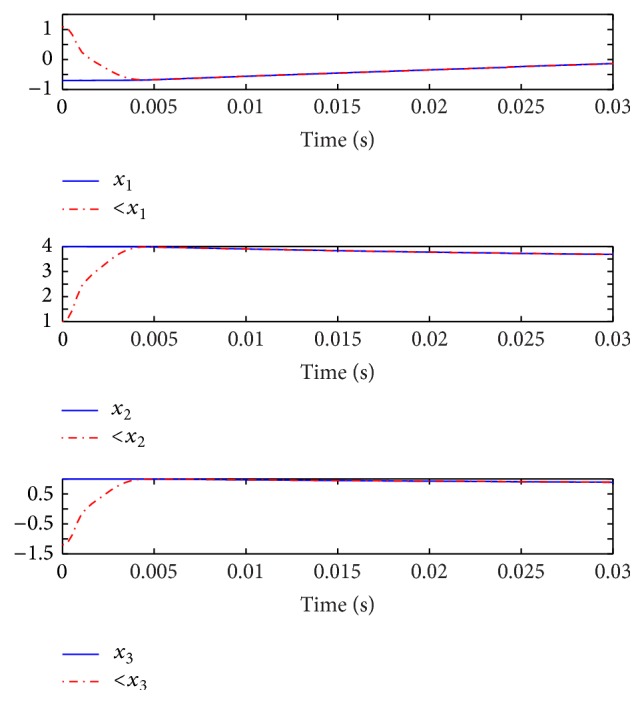
State responses of both master and slave systems.

**Figure 11:**
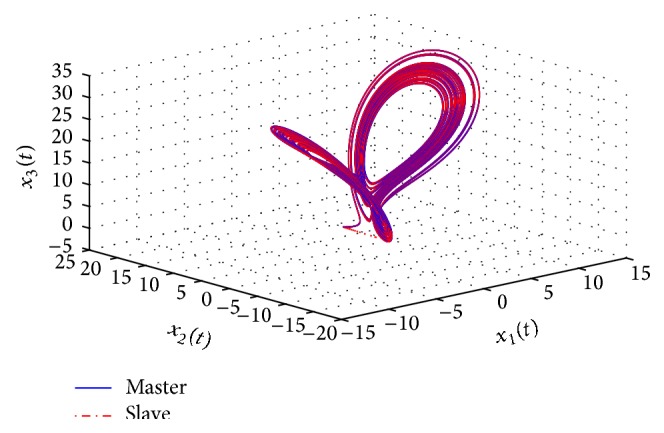
The chaotic behaviors of the master and the slave systems.

**Figure 12:**
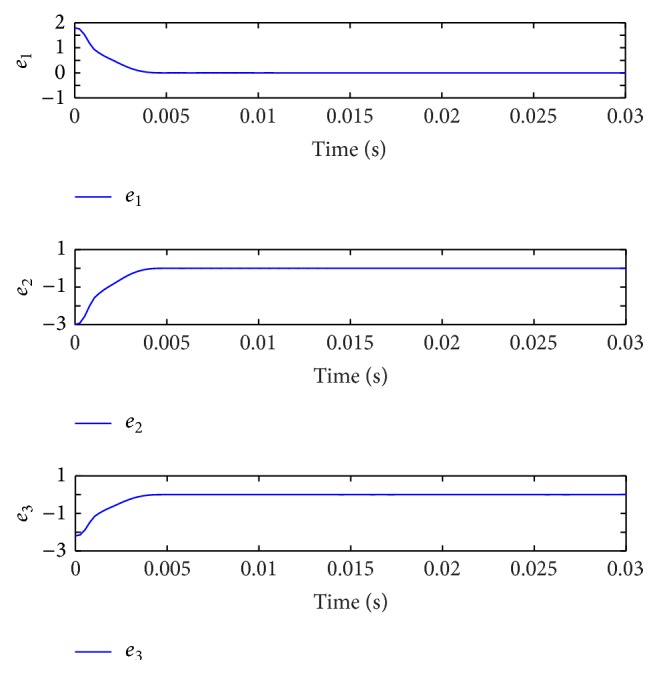
State responses of the error system.

**Figure 13:**
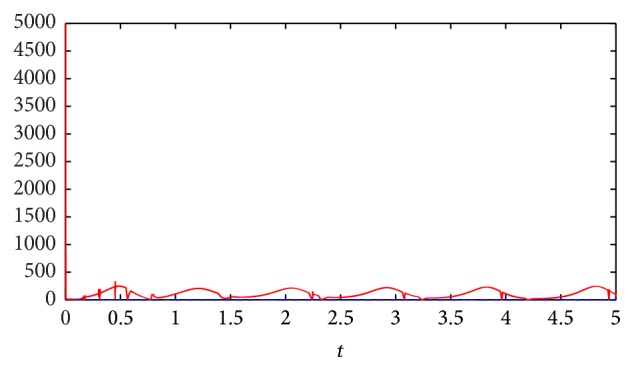
Plots of ‖Φ(*t*)‖ (blue line) and ‖∑_*i*=1_^8192^∑_*l*=1_^2^ *h*_*l*_(*t*)*h*_*i*_(*t*)Δ*R*_*il*_ *E*(*t*)‖ (red line).

Figure 10 displays the state responses of both the master and slave systems. The chaotic behaviors of the master and slave systems are shown in Figure 11. Moreover, Figure 12 illustrates the synchronization errors (*e*_1_, *e*_2_ and *e*_3_) which converge to zero. Furthermore, the assumption of ‖Φ(*t*)‖ ≤ ‖∑_*i*=1_^8192^∑_*l*=1_^2^ *h*_*i*_(*t*)*h*_*l*_(*t*)Δ*R*_*il*_ *E*(*t*)‖ is satisfied from the illustration shown in Figure 13.

*Step 9*. When the slave system synchronizes with the master system, we can retrieve the original message (Figure 13) from the output error signal and the decryption function.

The corresponding decryption function is the same as the encryption function:

(96) st=πι−t=F⋯FF︸nζι−t,−ϑt,−ϑt,…,−ϑt︸n,

where $\overset{-}{\iota }(t)$ is the output message (output error *Y*_*e*_(*t*)), *ϑ*(*t*) = 6 is the decryption key, and

(97) $$\begin{array}{c} F\left(s\left(t\right),\vartheta \left(t\right)\right)=\left\{\begin{array}{cc} \left(s\left(t\right)+\vartheta \left(t\right)\right)+1, & -1\leq \left(s\left(t\right)+\vartheta \left(t\right)\right)\leq -0.5\\ \left(s\left(t\right)+\vartheta \left(t\right)\right), & -0.5<\left(s\left(t\right)+\vartheta \left(t\right)\right)<0.5\\ \left(s\left(t\right)+\vartheta \left(t\right)\right)-1, & 0.5\leq \left(s\left(t\right)+\vartheta \left(t\right)\right)\leq 1. \end{array}\right. \end{array}$$

Figure 15 illustrates recovered error of the message *s*(*t*). Finally, the simulation results demonstrate that the exponential *H* ^*∞*^ synchronization of MTDC secure communication systems can recover the transmitted message by the designed fuzzy controller.

## 6. Conclusion 

**Figure 14:**
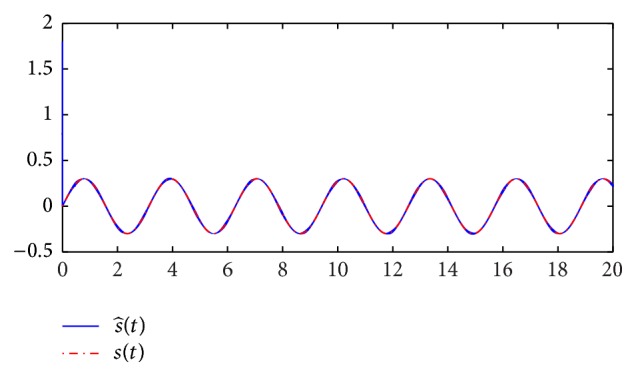
Recovered message $\overset{-}{s}(t)$ .

**Figure 15:**
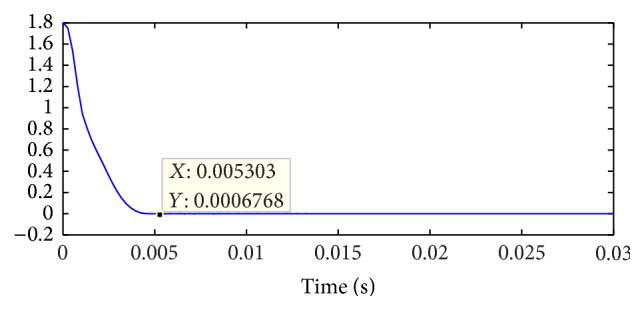
Recovered error of the message *s*(*t*).

In this paper, exponential synchronization multiple time-delay chaotic (MTDC) systems with optimal *H* ^*∞*^ performance and cryptography were combined to achieve a more secure communication system. First, we applied the *n*-shift cipher and key to the original message of transmission for encryption. The encrypted message is reencrypted using chaotic synchronization. The MTDC systems were then approximated using an NN model-based approach. Next, a robust model-based fuzzy control design was proposed to overcome the effect of modeling error between the MTDC systems and the NN models. In terms of Lyapunov's direct method, a delay-dependent stability criterion was derived to ensure that the slave system was able to exponentially synchronize with the master system. Subsequently, the stability conditions of this criterion were reformulated into linear matrix inequalities (LMIs). On the basis of the LMIs, a model-based fuzzy controller was then synthesized to stabilize the MTDC systems. Due to the capability of GA in random search for global optimization, the lower bound and upper bound of the search space can be set so that the GA will seek better feedback gains of fuzzy controllers in order to speed up the synchronization based on the feedback gains via LMI-based approach. Furthermore, according to the IGA which is demonstrated to have better performance than that of a traditional GA, we synthesized a fuzzy controller to realize the exponential *H* ^*∞*^ synchronization of the chaotic master-slave systems and reduce the *H* ^*∞*^ *-*norm from disturbance to synchronization error at the lowest level. On the other hand, the output error of the recovered message was stated using the *n*-shift cipher and key (Figure 14). Finally, the simulation results demonstrated that the exponential *H* ^*∞*^ synchronization of two different MTDC systems can be achieved by the designed fuzzy controller.

## Figures and Tables

**Table 1:** Specifications for IGA.

Population size	32

Number of generations	500

Coding of chromosome	Real-numbered string

Fitness function	Equation (31) with *t*_*f*_ = 600, *s*_*k*_ = *L*(*x*) in (63a), and pv = −10

Method of reproduction	Roulette wheel selection

Method of crossover	Improved crossover equations (26)–(29) with *w* = 0.3

Probability of mutation (*P*_*m*_)	0.08

**Table 2:** IGA experiments with various *w* and *P*_*m*_.

*w*	*P*_*m*_									
	0.01	0.02	0.03	0.04	0.05	0.06	0.07	0.08	0.09	0.1
0.9	0.6631	0.6639	0.6645	0.623	0.6626	0.663	0.6628	0.6626	0.6619	0.663
0.8	0.6598	0.6633	0.6647	0.6641	0.6635	0.6643	0.6631	0.6633	0.659	0.6611
0.7	0.664	0.6643	0.6636	0.662	0.6638	0.6631	0.6599	0.6628	0.6627	0.6625
0.6	0.6623	0.6638	0.6617	0.6623	0.6619	0.6633	0.6595	0.661	0.6612	0.6592
0.5	0.6599	0.6631	0.6642	0.6632	0.6627	0.6621	0.6623	0.6634	0.6635	0.6633
0.4	0.6613	0.6625	0.6595	0.6644	0.6637	0.6629	0.6618	0.6593	0.6631	0.6614
0.3	0.6621	0.6643	0.6623	0.6634	0.6643	0.6635	0.6635	0.6644	0.6629	0.6618
0.2	0.6644	0.6637	0.6617	0.6613	0.6627	0.6643	0.6628	0.6618	0.6599	0.6628
0.1	0.6623	0.6644	0.6628	0.661	0.6611	0.662	0.6598	0.6621	0.6611	0.6635

## Nomenclature

*N*_*m*_: Master system
*N*_*s*_: Slave system
*X*(*t*): State vector of the master system
$\hat{X}(t)$: State vector of the slave system
*X*(*t* − *τ*_*k*_) (*k* = 1,2,…, *m*): State vector with delay
*s*(*t*): Plaintext
*ζ*(·): Encryption function
*π*(·): Decryption function
*ϑ*(*t*): Decryption key
*ι*(*t*): The encrypted signal
$\overset{-}{\iota }(t)$: The recovered decryption signal
*E*(*t*): State vector of the error system
*E*(*t* − *τ*_*k*_) (*k* = 1,2,…, *m*): State vector with delay of the error system
*τ*_*k*_ (*k* = 1,2,…, *m*): Time delays
*W* ^*σ*^: The weight matrix for the *σ*th layer
*v*_*ς*_^*σ*^(*t*) (*ς* = 1,2,…, *J* ^*σ*^; *σ* = 1,2,…, *S*): Net input of the *ς*th neuron, *σ*th layer
*T*(*v*_*ς*_^*σ*^(*t*)): The transfer function of the *ς*th neuron, *σ*th layer
${\tilde{A}}_{i}\;\;(i=1,2,\ldots ,\phi )$: Constant matrices
*g*_*ς*1_^*σ*^: The minimum derivative of *T*(*v*_*ς*_^*σ*^(*t*))
*g*_*ς*2_^*σ*^: The maximum derivative of *T*(*v*_*ς*_^*σ*^(*t*))
*G* ^*σ*^: The min-max matrix
*b*_*ς*_ (*ς* = 1,2,…, *J*): The variables *φ* of the *ς*th neuron of the first layer
*n*_*ς*_ (*ς* = 1,2,…, *J*): The variables *φ* of the *ς*th neuron of the second layer
*p*_*ς*_ (*ς* = 1,2,…, *J*): The variables *φ* of the *ς*th neuron of the *S*th layer
os_*c*_^1^ ~ os_*c*_^4^: The chromosomes of the next generation
*P*_1_ and *P*_2_: The two chromosomes chosen from the parents
Fit(Λ): The fitness value of the Λth chromosome in a population
*e*_*η*_^Λ^(*t*): The error of the Λth chromosome in a population
pv: A punishing value
*P*: A symmetric positive definite matrix
*Q*: A symmetric positive definite matrix satisfies *Q* = *P* ^−1^.
